# Supplementary material for: Gender-Specific Prognostic Impact of Treosulfan Levels in High-Dose Chemotherapy for Multiple Myeloma
Source: Cancers (Basel). 2024 Oct 1;16(19):3364. doi: 10.3390/cancers16193364 (PMC11475554; doi:10.3390/cancers16193364)
Supplement: Supplementary file 1 [file cancers-16-03364-s001.zip › cancers-3224199-supplementary.pdf]

**Table S1.** Multivariate analysis of progression free survival.

| **Table S1: Cox-proportional hazard model for progression free survival** | | | | | | |
|---|---|---|---|---|---|---|
| | | **Univariate** | | | **Multivariable** | |
| **Characteristic** | **HR** | **95% CI** | **p-value** | **HR** | **95% CI** | **p-value** |
| Age | 1.01 | 0.96, 1.06 | 0.6 | 1.00 | 0.93-1.06 | >0.9 |
| Gender | | | | | | |
| Male | — | — | | — | — | |
| Female | 0.57 | 0.21- 1.55 | 0.3 | 0.35 | 0.11 1.13 | 0.078 |
| Cytogenetic risk group | | | | | | |
| Normal | — | — | | — | — | |
| High | 4.02 | 1.67-9.69 | 0.002 | 8.75 | 2.55-30.1 | <0.001 |
| Remission status before HDCT/ASCT | | | | | | |
| CR | — | — | | — | — | |
| VGPR | 1.21 | 0.34-4.34 | 0.8 | 0.92 | 0.24-3.51 | >0.9 |
| PR | 0.91 | 0.23-3.65 | 0.9 | 0.77 | 0.16-3.58 | 0.7 |
| SD | 2.36 | 0.24-23.2 | 0.5 | 4.25 | 0.33-54.6 | 0.3 |
| AUC of Treosulfan levels [mg*h/L] | | | | | | |
| < 900 | — | — | | — | — | |
| > 900 | 2.04 | 0.84-4.93 | 0.11 | 1.96 | 0.64-5.99 | 0.2 |
| Peak levels of Treosulfan [mg/L] | | | | | | |
| < 400 | — | — | | — | — | |
| > 400 | 2.05 | 0.69-6.13 | 0.2 | 4.57 | 0.96-21.9 | 0.057 |

**Table S2.** Multivariate analysis of progression free survival.

| **Table S2: Cox-proportional hazard model for overall survival** | | | | | | |
|---|---|---|---|---|---|---|
| | | **Univariate** | | | **Multivariable** | |
| **Characteristic** | **HR** | **95% CI** | **p-value** | **HR** | **95% CI** | **p-value** |
| Age | 1.00 | 0.93-1.07 | >0.9 | 0.97 | 0.88-1.07 | 0.5 |
| Gender | | | | | | |

| | | | | | | |
|---|---|---|---|---|---|---|
| Male | — | — | | — | — | |
| Female | 1.12 | 0.28-4.50 | 0.9 | 0.46 | 0.07-2.87 | 0.4 |
| Cytogenetic risk group | | | | | | |
| Normal | — | — | | — | — | |
| High | 6.12 | 1.53-24.5 | 0.011 | 87.3 | 3.94-1935 | 0.005 |
| Remission status before HDCT/ASCT | | | | | | |
| CR | — | — | | — | — | |
| VGPR | 1.86 | 0.22-15.5 | 0.6 | 0.88 | 0.09-8.49 | >0.9 |
| PR | 0.43 | 0.03-6.97 | 0.6 | 0.33 | 0.02-6.90 | 0.5 |
| SD | 4.68 | 0.28-79.4 | 0.3 | 52.6 | 1.09-2543 | 0.045 |
| AUC of Treosulfan levels [mg*h/L] | | | | | | |
| <900 | — | — | | — | — | |
| >900 | 3.51 | 0.89-13.8 | 0.073 | 2.89 | 0.41-20.5 | 0.3 |
| Peak levels of Treosulfan [mg/L] | | | | | | |
| <400 | — | — | | — | — | |
| >400 | 4.35 | 1.08-17.5 | 0.039 | 33.0 | 1.65-658 | 0.022 |
